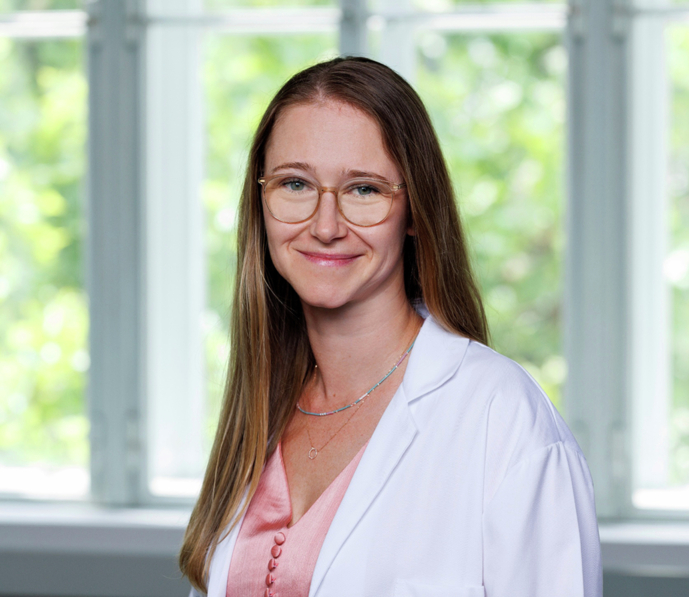# Katharina Goeral: ECI biocommentary

**DOI:** 10.1038/s41390-026-04887-8

**Published:** 2026-03-09

**Authors:** Katharina Goeral

**Affiliations:** Department of Pediatrics and Adolescent Medicine - Division of Neonatology, Intensive Care and Pediatric Neurology, Medical University of Vienna, Comprehensive Center for Pediatrics, full Member of ERN EpiCare, Vienna, Austria

Caring for extremely preterm and critically ill newborns requires life-altering decisions within hours or days, often with limited objective information about their neurological future. At the bedside, families ask questions about long-term outcomes that we frequently cannot answer with confidence. Experiencing this uncertainty early in my training became the central motivation for my research: I sought to replace subjective estimates with measurable, biologically grounded predictors that can meaningfully guide clinical care.

I trained in pediatrics and neonatology at the Medical University of Vienna and completed a PhD in Clinical Neurosciences focused on neonatal brain injury. An early research period at the Kennedy Krieger Institute and Johns Hopkins Medical Institutions introduced me to translational neuroscience and mechanistic models of perinatal brain damage, shaping my view that clinical problems must be addressed through integrated biology, physiology, and technology. Since then, my work has consistently aimed to bridge bedside neonatology with quantitative and data-driven approaches.

My research program advances precision medicine for vulnerable newborns by integrating multimodal biomarkers, neurophysiology, and advanced neuroimaging. I lead interdisciplinary collaborations that combine biomarkers in blood, urine, and CSF (proteomics and metabolomics) with amplitude-integrated EEG, near-infrared spectroscopy, quantitative MRI, and, increasingly, neonatal hemodynamics using targeted echocardiography. Supported by competitive funding, these efforts have enabled the establishment of biobanks and multimodal datasets analyzed with machine-learning approaches to identify early injury patterns, improve risk stratification, and support individualized decision-making. The overarching goal is to translate complex biological information into decisions that matter at the bedside.

Working in a tertiary referral NICU continuously shapes my scientific questions and ensures that our projects address real clinical needs. I also mentor a growing group of PhD students and young clinician-scientists, fostering collaborations across neonatology, radiology, and data sciences.

Being recognized as an Early Career Investigator by *Pediatric Research* is both an honor and an encouragement to continue pursuing this interdisciplinary path. It reinforces my commitment to moving neonatal monitoring from episodic assessments toward continuous, integrated, and predictive precision medicine. By linking brain health with real-time physiology, I hope not only to improve prognostication but increasingly to anticipate vulnerability, prevent injury, and improve lifelong neurodevelopmental outcomes for our smallest patients and their families.